# Disease spectrum of abnormal serum free light chain ratio and its diagnostic significance

**DOI:** 10.18632/oncotarget.19391

**Published:** 2017-07-19

**Authors:** Bin Xu, Yi Tang, Jianfeng Zhou, Peiling Zhang, Huijun Li

**Affiliations:** ^1^ Department of Hematology, Tongji Hospital, Tongji Medical College, Huazhong University of Science and Technology, Wuhan, 430030, China; ^2^ Department of Clinical Laboratory, Tongji Hospital, Tongji Medical College, Huazhong University of Science and Technology, Wuhan 430030, China

**Keywords:** abnormal serum free light chain ratio, cut-off value, malignant plasma diseases, MGUS, reactive plasma diseases

## Abstract

**Objective:**

To analyze the spectrum of abnormal serum free light chain ratio (sFLC κ/λ ratio), and to redefine the range of sFLC κ/λ ratio, so as to achieve hierarchical diagnosis of diseases with abnormal sFLC κ/λ ratio, resulting in the increased sensitivity and specificity in the diagnosis of monoclonal plasma diseases.

**Methods:**

Enrolled 1,340 patients with abnormal sFLC κ/λ ratio (<0.26 or >1.65) were grouped: (1) group A: malignant plasma diseases; (2) group B: monoclonal gammopathies of undetermined significance (MGUS); (3) group C: reactive plasma diseases. These patients were further divided by renal function eGFR <60 or >60 ml/min/1.73m^2^ to eliminate renal diseases influencing the results. Statistical analyses was performed by using SPSS 22 software.

**Results:**

When sFLC κ/λ ratio >3.49 and eGFR >60ml/min/1.73m^2^, the sensitivity and specificity of the diagnosis of malignant plasma diseases were 86.1% and 94.0%, respectively. When sFLC κ/λ ratio >2.89 and eGFR <60ml/min/1.73m^2^, the sensitivity and specificity of the diagnosis of malignant plasma diseases were 92.0% and 97.0%, respectively.

**Conclusion:**

The sensitivity and specificity of the diagnosis of monoclonal plasma diseases can be significantly improved by redefining the cut-off value of sFLC κ/λ ratio and the renal function index of eGFR.

## INTRODUCTION

Serum free light chain (sFLC) assay (Freelite) is an antibody-based system that measures kappa and lambda immunoglobulin light chains unbound to heavy chains in serum. The measurement of sFLC and k/λ ratio, with adequate sensitivity and specificity for the detection of excess monoclonal sFLC [1], is useful for the diagnosis, monitoring, and prognosis prediction of monoclonal gammopathies (MGs) [2].

In clinical practice, plasma diseases contain multiple systemic diseases: multiple myeloma, POEMS syndrome, primary systemic amyloidosis (Kidney, heart, skin, intestine, etc.). Therefore, in different clinical departments, when patients have different clinical manifestations, highly suspected or need to exclude monoclonal plasma diseases, the detection of serum free light chain (sFLC), serum protein electrophoresis (SPE) and serum immunofixation electrophoresis (sIFE) will be all completed. Based on the results of the test, whether the patient needs to improve the bone marrow aspiration, biopsy of local tissues and other examinations will be determined subsequently to further clarify the diagnosis.

The monoclonal plasma diseases may have a variety of clinical manifestation, which may be shown as abnormal hemogram, nervous system disorder, cardiac muscle or kidney amyloidosis and skeleton destroy, etc. Generally, the clinical physician would think that, the sFLC ratio anomaly normally prompts the monoclonal plasma diseases, especially when the M protein is detected as negative. It is of great importance to employ the sFLC assay to assist the diagnosis.

According to Guenet et al.’s report, the normal reference range for sFLC k/λ ratio is [0.26, 1.65], and for one with a value beyond this range the presence of monoclonal plasma diseases should be considered [3]. However, in the clinical practice, abnormal sFLC k/λ ratios are frequently noted in cases with evidence supporting no MGs but reactive disorders, eg. auto-immune diseases and chronic nephrosis. To date, the clinical relevance and implications of sFLC k/λ ratio have not been fully investigated. Before a clear understanding can be established, several issues should be addressed. First, it should be determined the whole spectrum of diseases possibly presenting deviated sFLC k/λ ratios. Second, further evaluation and elaboration with adequate sample size is needed to locate better cut-off values for the diagnosis of MGs. To this end, we conducted this retrospective study involving a large group of individuals.

## RESULTS

### Spectrum of diseases with an abnormal sFLC ratio

Among 4,786 patients accepted the sFCL assay in preliminary diagnosis, a total of 1,340 patients with combined abnormal sFLC ratio is screened out. See Table 1 for the specific diseases spectrum distribution of 1,340 cases. Of which, there are the multiple myeloma (MM, 310 cases, 23.1%), plasma cell leukemia (PCL, 8 cases, 0.6%), Waldenstrommacroglobulinemia/ lymphoplasmacytic lym-phoma (WM/LPL, 23 cases, 1.7%), solitary plasmacytoma (SP, 7 cases, 0.5%), primary systematic amyloidosis (AL, 15 cases, 1.1%), B cell non-Hodgkin’s lymphoma (B-NHL, 19 cases, 1.4%), monoclonal gamnopathy of undetermined significance (MGUS, 62 cases, 4.6%), autoimmune disease (AID, 104 cases, 7.8%), chronic kidney disease (KD, 562 cases, 41.9%), infection/chronic inflammatory condition (I/CIC, 76 cases, 5.7%), non-lymphoid neoplasia (NLN, 40 cases, 3.0%), peripheral neuropathy (PN, 11 cases, 0.8%), myocardial lesions (HD, 23 cases, 1.7%) and other benign diseases (OBD, 74 cases, 5.5%). There are 835 male patients (62.3%) and 505 female patients (37.3%). Median onset age is 58 years old (14-94 years old), and 891 (66.5%) patients are over 50 years old, while about 450 (33.6%) patients are diagnosed as monoclonal plasma diseases.

**Table 1:** Clinical characteristics and diagnoses in diseases with abnormal sFLC ratio

Disease group	Cases (N)	Cases (%)	Male (N)	Female (N)	Age (Median)	Age (Range)	Age>50 (N)	Age>50 (%)
MM	310	23.1%	210	100	58	32-84	234	73.6%
PCL	8	0.6%	7	1	54	42-66	5	62.5%
WM/LPL	23	1.7%	20	3	64	43-76	19	82.6%
SP	7	0.5%	6	1	47	29-57	1	14.3%
POEMS	6	0.5%	2	4	49.5	33-59	2	33.3%
AL	15	1.1%	10	5	53	25-74	8	53.3%
B-NHL	19	1.4%	10	9	61	22-82	14	73.7%
MGUS	62	4.6%	37	25	61	14-84	45	72.6%
AID	104	7.8%	42	62	54	19-82	58	55.8%
KD	562	41.9%	353	209	55	18-93	341	60.7%
I/CIC	76	5.7%	48	28	62	17-94	57	75.0%
NLN	40	3.0%	25	15	59	22-83	28	70.0%
PN	11	0.8%	8	3	55	38-78	7	63.6%
HD	23	1.7%	14	9	65	19-83	18	78.3%
OBD	74	5.5%	47	27	63.5	20-89	59	79.7%
Total	1340	100.0%	835	505	58	14-94	891	66.5%

MM, multiple myeloma; PCL, plasma cell leukemia; WM, Waldenstrom macroglobulinemia; LPL, lymphoplasmacytoid lymphoma; SP, solitary plasmacytoma; POEMS, POEMS syndrome; AL, primary amyloidosis; B-NHL, B cell non-Hodgkin’s lymphoma; MGUS, monoclonal gammopathy of udetermined significance; AID, autoimmune disease; KD, kidney disease; I/CIC, Infection/chronic inflammatory condition; NLN, Non-lymphoid neoplasia; PN, peripheral neuropathy; HD, heart disease: myocardiopathy; OBD, other benign diseases; Other diseases mainly contained cerebrovascular diseases and endocrine metabolism system diseases.

### Disease profiles

In the disease spectrum of abnormal sFLC ratio as shown in Table 3, the malignant plasma diseases occupy 29.0%, with MGUS of 4.6%, and reactive plasma diseases of 66.4%. The analysis result is basically same as the conclusion published on American Journal of Clinical Pathology [8].

**Table 2:** The distribution of the diseases with different range of sFLC ratio

Disease	Cases	rFLC<0.26	rFLC<0.26	rFLC<0.26	rFLC>1.65	rFLC>1.65	rFLC>1.65
	(N)	(N)	(%)	(median)	(N)	(%)	(median)
MM	310	147	75.4%	0.05	163	14.2%	35.32
PCL	8	5	2.6%	0.04	3	0.3%	16.81
WM/LPL	23	5	2.6%	0.08	18	1.6%	19.90
SP	7	1	0.5%	0.03	6	0.5%	33.57
POEMS	6	5	2.6%	0.13	1	0.1%	5.24
AL	15	11	5.6%	0.12	4	0.3%	10.40
B-NHL	19	4	2.1%	0.16	15	1.3%	10.93
MGUS	62	11	5.6%	0.15	51	4.5%	6.52
AID	104	0	0.0%	N	104	9.1%	2.05
KD	562	3	1.5%	0.11	559	48.8%	2.37
I/CIC	76	2	1.0%	0.16	74	6.5%	2.12
NLN	40	0	0.0%	N	40	3.5%	2.19
PN	11	0	0.0%	N	11	1.0%	2.02
HD	23	0	0.0%	N	23	2.0%	2.12
OBD	74	1	0.5%	0.10	73	6.4%	2.04
Total	1340	195	100.0%	0.06	1145	100.0%	7.22

MM, multiple myeloma; PCL, plasma cell leukemia; WM, Waldenstrom macroglobulinemia; LPL, lymphoplasmacytoid lymphoma; SP, solitary plasmacytoma; POEMS, POEMS syndrome; AL, primary amyloidosis; B-NHL, B cell non-Hodgkin’s lymphoma; MGUS, monoclonal gammopathy of undetermined significance; AID, autoimmune disease; KD, kidney disease; I/CIC, Infection/chronic inflammatory condition; NLN, Non-lymphoid neoplasia; PN, peripheral neuropathy; HD, heart disease: myocardiopathy; OBD, other benign diseases; Other diseases mainly contained cerebrovascular diseases and endocrine metabolism system diseases; rFLC, the ratio of sFLC.

**Table 3:** The distribution of different groups of the diseases with abnormal sFLC ratio

Disease group	Abnormal rFLC	Abnormal rFLC	Abnormal rFLC
	<0.26 or >1.65	<0.26	>1.65
A(n, n%)	388(29.0%)	178(91.3%)	210(18.3%)
B(n, n%)	62(4.6%)	11(5.6%)	51(4.5%)
C(n, n%)	890(66.4%)	6(3.1%)	884(77.2%)
A+B(n, n%)	450(33.6%)	189(96.9%)	261(22.8%)
A+B+C(n, n%)	1340(100.0%)	195(100.0%)	1145(100.0%)

Group A, malignant plasma diseases; Group B, MGUS; Group C, reactive plasma diseases; rFLC, the ratio of serum free light chain.

Tables 2 and 3 show: (1) When sFLC κ/λ ratio<0.26, MM occupies 29.0%, which is the main disease type within this interval range. Malignant plasma diseases take up about 91.3%, with MGUS about 75.4% and reactive plasma diseases about 3.1%. This part of benign diseases mainly includes 3 patients with chronic kidney disease, 2 patients with infectious disease and 1 diabetic patients. (2) When sFLC κ/λ ratio>1.65, the patients with chronic kidney disease occupies about 48.8%, which is the main disease type within this interval range. Malignant plasma diseases take up about 48.8%, with MGUS about 4.5% and reactive plasma diseases about 77.2%. The cases of malignant diseases mainly comprise 163 patients with MM, 3 patients with PCL, 18 patients with WM/LPL, 6 patients with SP, 1 patient with POEMS, 4 patients with AL and 15 patients with B-NHL.

In addition, among 4,786 patients, there are 143 patients with normal sFLC ratio, concomitant with monoclonal plasma diseases of positive M protein in SPE and sIFE, and see Table 4 for the details. Its disease spectrum composition mainly is: MM (27.3%), PCL (0), WM/LPL (1.4%), SP (3.5%), POEMS (9.1%), AL (1.4%), B-NHL (4.2%) and MGUS (53.2%).

**Table 4:** Comparison between MGs with abnormal sFLC ratio and MGs with normal sFLC ratio

Disease	Case (N)	Case (%)	Case (N)	Case (%)	Total (N)
	Abnormal sFLC ratio		Normal sFLC ratio		
MM	310	68.9%	39	27.3%	349
PCL	8	1.8%	0	0.0%	8
WM/LPL	23	5.1%	2	1.4%	25
SP	7	1.6%	5	3.5%	12
POEMS	6	1.3%	13	9.1%	19
AL	15	3.3%	2	1.4%	17
B-NHL	19	4.2%	6	4.1%	25
MGUS	62	13.8%	76	53.2%	138
Total	450	100.0%	143	100.0%	593

MGs, monoclonal gammopathies; MM, multiple myeloma; PCL, plasma cell leukemia; WM, Waldenstrom macroglobulinemia; LPL, lymphoplasmacytoid lymphoma; SP, solitary plasmacytoma; POEMS, POEMS syndrome; AL, primary amyloidosis; B-NHL, B cell non-Hodgkin’s lymphoma; MGUS, monoclonal gammopathy of undetermined significance.

To compare the disease spectrum of monoclonal plasma diseases in accordance with the normal and abnormal groups of sFLC ratio, the result in Table 4 indicates: there are 310 patients with MM of abnormal sFLC ratio, but 39 patients with MM of normal sFLC ratio; 15 patients with AL of abnormal sFLC ratio, but 2 AL of normal sFLC ratio; 62 patients with MGUS of abnormal sFLC ratio, but 76 patients with normal sFLC ratio.; 6 patients with POEMS of abnormal sFLC ratio, but 13 patients with POEMS of normal sFLC ratio.

### Diagnostic value of the levels of sFLC k/λ ratio

Among 1,340 patients with abnormal sFLC ratio, there are 50 patients whose combined sFLC κ/λ ratio <0.01, including 46 patients with MM, 3 patients with PCL and 1 patient with WM/LPL; there are 41 patients whose combined sFLC κ/λ ratio>100, all of which get the multiple myeloma.

**Figure 1:**
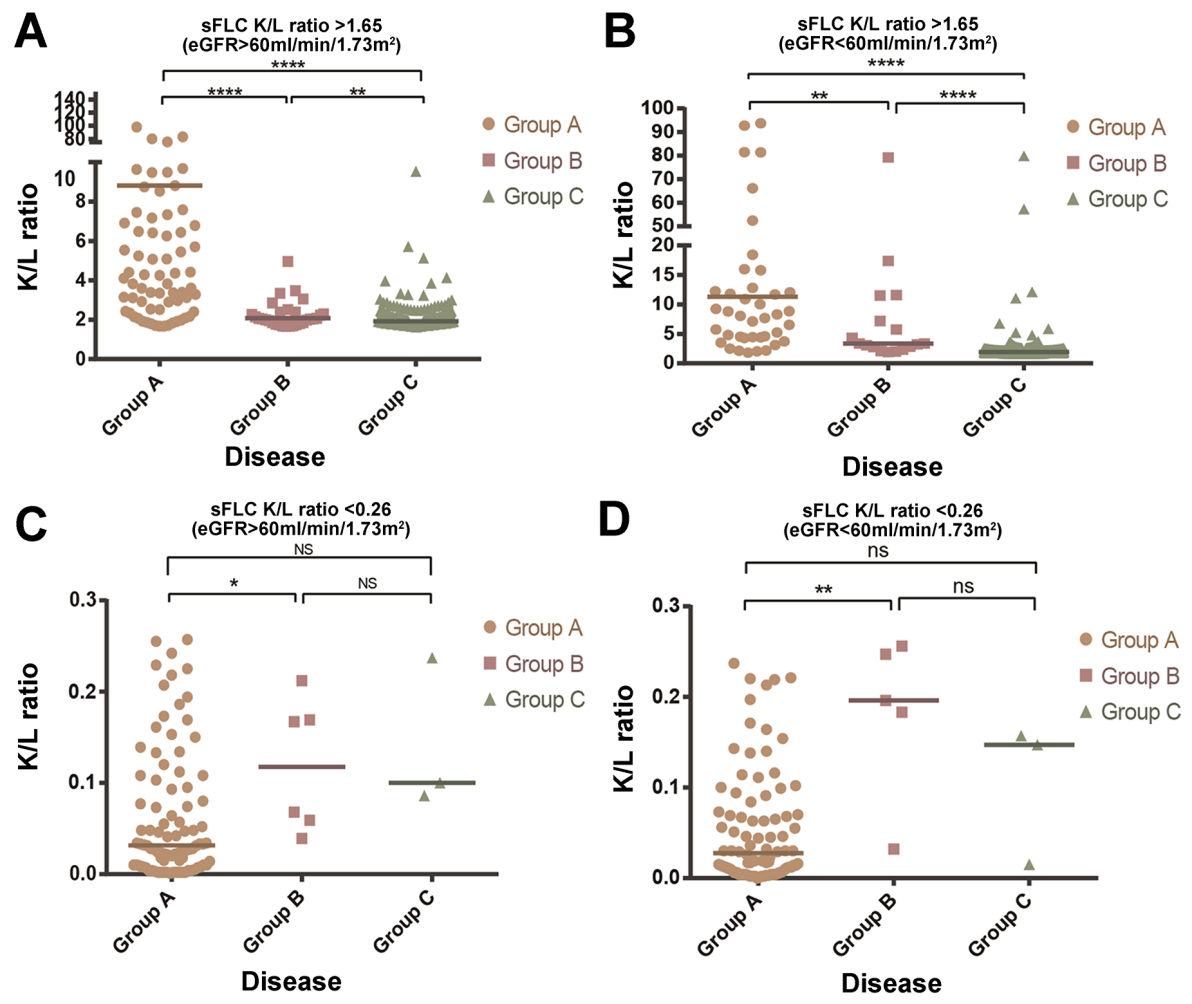
Numerical difference of sFLC ratio among three groups (Group A, B, C) (A) sFLC K/L ratio>1.65 and eGFR>60ml/min/1.73m^2^ (B). sFLC K/L ratio>1.65 and eGFR<60ml/min/1.73m^2^ (C). sFLC K/L ratio<0.26 and eGFR>60ml/min/1.73m^2^ **(D)**. sFLC K/L ratio<0.26 and eGFR<60ml/min/1.73m^2^. Group A, Malignant plasma diseases; Group B, MGUS; Group C, Reactive plasma diseases. * Indicates P<0.05; ** indicates P<0.01; **** indicates P<0.0001; ns Indicated no significance.

When sFLC κ/λ ratio >1.65, no matter whether the patient has been combined with renal damage, the sFLC ratio difference of Group A, B and C have obvious statistics meaning (P<0.05). See Figure 1A and Figure 1B for the details. When sFLC κ/λ ratio <0.26, no matter whether the patient has been combined with renal damage, the sFLC ratio difference between Group A and C, or between Group B and C have no obvious statistics meaning (P>0.05), but the sFLC ratio difference between Group A and B have statistics meaning (P<0.05), See Figure 1C and 1D for the details.

When sFLC κ/λ ratio <0.26, although the exact number of cases in different groups of group A, group B and group C were significantly different, corresponding statistical analysis results suggested that there were 189 cases in group A+B, and 6 cases in group C; meanwhile, when sFLC κ/λ ratio <0.26, 96.9% of the patients were diagnosed as monoclonal plasma diseases, only the remaining 3.1% of patients were reactive plasma diseases. Furthermore, the correct diagnostic rate of monoclonal plasma diseases was high when sFLC κ/λ ratio <0.26. The above investigation results might have a good guiding significance for clinical diagnosis. Therefore, the present study focused on the redefining the new critical value of sFLC κ/λ ratio when the ratio greater than 1.65, so as to improve the correct diagnostic rate of monoclonal plasma diseases.

As for 4,786 cases incorporated into the sFLC screening, by statistics analysis, the sensitivity and specificity of diagnosis test is calculated by applying the fourfold table method, with statistic result showing: the sensitivity and specificity of abnormal sFLC ratio to judge malignant plasma diseases respectively are 85.27% and 78.02%. See Table 5 for the details.

**Table 5:** Assessment of sFLC assay for diagnosing monoclonal gammopathies

sFLC assay	MGs	Non-MGs	Total patients
	n	n	
abnormal rFLC	450(a)	890(b)	1340(a+b)
normal rFLC	143(c)	3303(d)	3446(c+d)
total patients	593(a+c)	4193(b+d)	4786(a+b+c+d)
sFLC assay for diagnosing MGs			
Sensitivity	85.3%(a/a+c)	false positive rate	22.0%(b/b+d)
Specificity	78.0%(d/b+d)	false negative rate	14.7%(c/a+c)
positive predictive value	33.6%(a/a+b)		
negative predictive value	95.9%(d/c+d)		

MGs, monoclonal gammopathies; Non-MGs, non-monoclonal gammopathies; normal rFLC, the ratio of sFLC is from 0.26 to 1.65; abnormal rFLC, the ratio of sFLC is <0.26 or >1.65.

**Figure 2:**
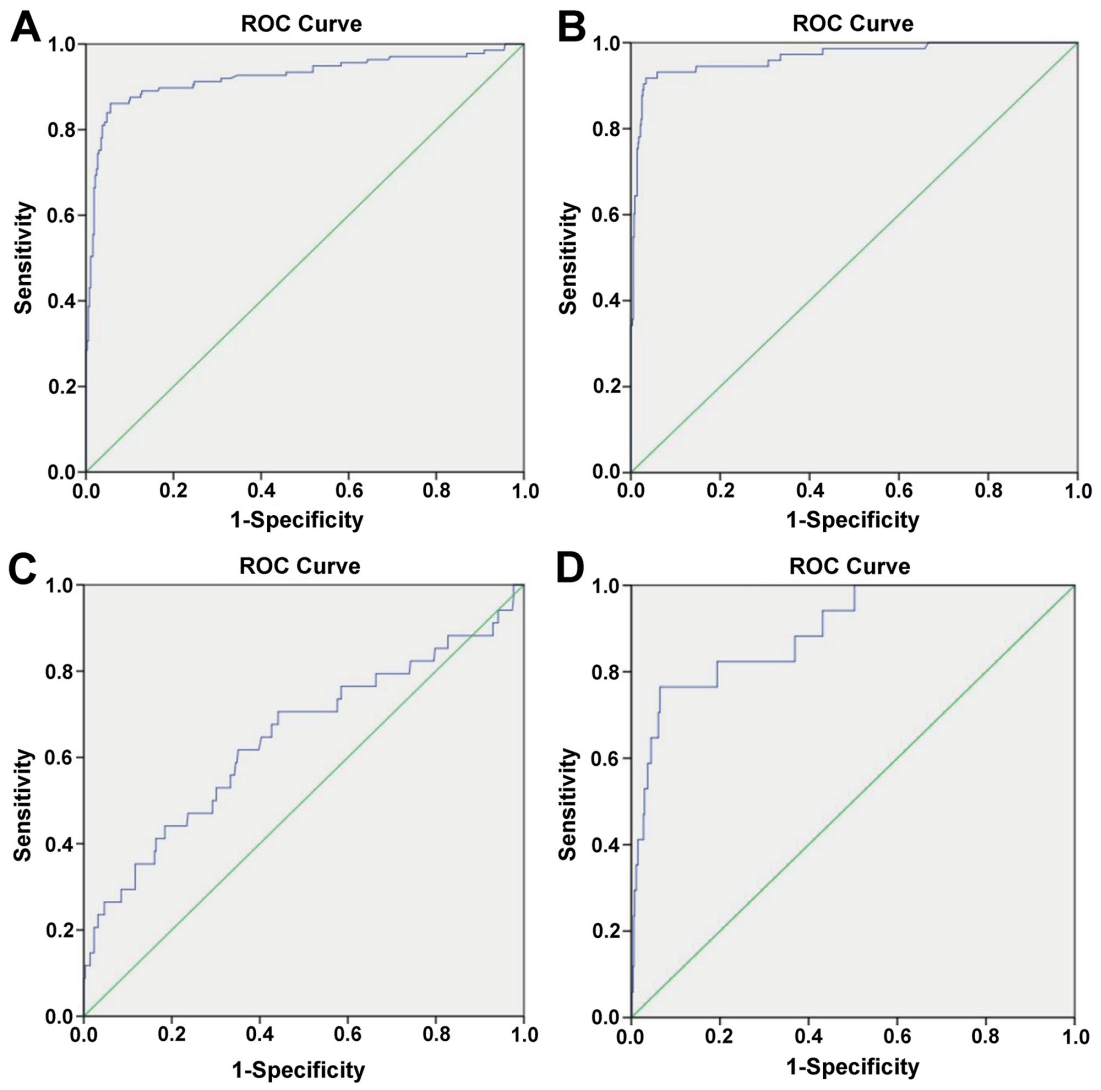
The ROC curve for diagnosing monoclonal gammopathies (MGs) **(A)** sFLC K/L ratio>1.65 and eGFR>60ml/min/1.73m^2^, the cut-off value of sFLC ratio is 2.89 for diagnosing malignant plasma diseases. **(B)** sFLC K/L ratio>1.65 and eGFR<60ml/min/1.73m^2^, the cut-off value of sFLC ratio is 3.49 for diagnosing malignant plasma diseases. **(C)** sFLC K/L ratio>1.65 and eGFR>60ml/min/1.73m^2^, the cut-off value of sFLC ratio is 2.03 for diagnosing MGUS. **(D)** sFLC K/L ratio>1.65 and eGFR<60ml/min/1.73m^2^, the cut-off value of sFLC ratio is 2.79 for diagnosing MGUS.

Therefore, the new threshold value of sFLC ratio of malignant plasma diseases and MGUS shall defined by applying ROC curve, so as to improve the sensitivity and specificity of diagnosing the monoclonal plasma diseases. In order to eliminate the influence of renal function damage to the sFLC ratio, we make further analysis on cases with sFLC κ/λ ratio >1.65, with statistics result as shown in Figure 2 and Table 6.

**Table 6:** Characteristics of new cut-off value of sFLC ratio for diagnosing MGs through ROC curve

	Group A		Group B	
sFLC ratio	>1.65	>1.65	>1.65	>1.65
eGFR (ml/min/1.73m^2^)	>60	<60	>60	<60
new cut-off value (sFLC ratio)	2.89	3.49	2.03	2.79
The area under ROC	0.924	0.966	0.644	0.893
95%CI	0.891-0.957	0.942-0.990	0.532-0.755	0.816-0.971
P value	<0.0001	<0.0001	0.006	<0.0001
sensitivity(%)	86.1%	92.0%	62.0%	77.0%
specificity(%)	94.0%	97.0%	65.0%	93.4%

Group A, malignant plasma diseases; Group B, MGUS; MGs, monoclonal gammopathies.

When sFLC κ/λ ratio >1.65 and eGFR >60ml/min/1.73m^2^, the optimal cut-off value for diagnosing the malignant plasma diseases is sFLC κ/λ ratio=2.89. The area under the ROC curve is 0.924, and 95% confidence interval is 0.891-0.957, with P<0.0001. In particular, when sFLC κ/λ ratio >2.89, the sensitivity and specificity for diagnosing the malignant plasma diseases respectively are 86.1% and 94.0%.

When sFLC κ/λ ratio >1.65 and eGFR <60ml/min/1.73m^2^, the optimal cut-off value for diagnosing the malignant plasma diseases is sFLC κ/λ ratio=3.49. The area under the ROC curve is 0.966, and 95% confidence interval is 0.942-0.990, with P<0.0001. In particular, when sFLC κ/λ ratio >3.49, the sensitivity and specificity for diagnosing the malignant plasma diseases respectively are 92.0% and 97.0%.

When sFLC κ/λ ratio >1.65 and eGFR >60ml/min/1.73m^2^, the optimal cut-off value for diagnosing MGUS is sFLC κ/λ ratio=2.03. The area under the ROC curve is 0.644, and 95% confidence interval is 0.532-0.755, with P=0.006 (P<0.05). In particular, when sFLC κ/λ ratio >2.03, the sensitivity and specificity for diagnosing MGUS respectively are 62.0% and 65.0%.

When sFLC κ/λ ratio >1.65 and eGFR <60ml/min/1.73m^2^, the optimal cut-off value for diagnosing MGUS is sFLC κ/λ ratio=2.79. The area under the ROC curve is 0.893, and 95% confidence interval is 0.816-0.971, with P<0.0001. In particular, when sFLC κ/λ ratio >2.79, the sensitivity and specificity for diagnosing MGUS respectively are 77.0% and 93.4%.

### MGUS with different comorbidities

For 62 MGUS patients screened out from 1,340 patients, the main comorbidities mainly consist of the chronic kidney diseases (40.3%), connective tissue diseases (6.5%), other autoimmune diseases (8.1%), infection/chronic inflammatory condition (19.4%), myocardiopathy (8.1%), peripheral neuropathy (8.1%) and non-lymphoid neoplasia (9.7%). The MGUS cases screened out in this study are the data information acquired from the treatment in the hospital due to various clinical manifestations, not the cases screened out by physical examination in the Physical Examination Center of our hospital. MGUS normally combines all kinds of concomitant diseases, which respectively are, in accordance with comorbidities from high to low: chronic kidney disease, infection/chronic inflammatory condition, autoimmune disease including connective tissue diseases, non-lymphoid neoplasia, peripheral neuropathy and myocardiopathy. Among 62 MGUS patients following up for 2 years, 1 IgM-Kappa MGUS patient concomitant with chronic nephrotic syndrome has progresses to be marginal zone lymphoma 3 months after the confirmed diagnosis. 2 IgG-Kappa MGUS patients concomitant with connective tissue diseases have progressed to be multiple myeloma 1 year after the confirmed diagnosis. Besides, for 12 MGUS patients concomitant with infection/chronic inflammatory condition, by actively controlling the infection or controlling the illness activity, the patients’ sFLC ratios get right after 3 months.

### Systemic amyloidosis

22 MGUS patients with combined eGFR <60ml/min/1.73m^2^ are further screened in this study. Although the part of patients has carried out the bone marrow puncture cytology and bone marrow biopsy, they do not accept the renal aspiration biopsy. The part of patients mainly maintains the treatment by hematodialysis, without giving chemotherapy and other immunoregulation treatment.

### POEMS syndrome

15 patients with primary amyloidosis of abnormal sFLC ratio are screened out in this clinical research, of which there are 4 patients with sFLC ratio >1.65 and 11 patients with sFLC ratio <0.26. By further combining the patients’ SPE, sIFE and uIFE detection results, it is found that, 7 patients are in free light chain lambda type, 3 in IgG-lambda type, 1 in IgA-lambda type, 2 IgM-kappa type and 2 IgG-kappa types. Among these 15 patients, 12 patients get combined single organ involvement (7 patients are manifested as renal amyloidosis and 5 patients are manifested as heart amyloidosis). All of the rest 3 patients are manifested as involvement of more than 2 organs.

In 6 POEMS patients with abnormal sFLC ratio, there is 1 patient with sFLC ratio >1.65, and 5 patients with sFLC ratio <0.26. Combined with the patients’ SPE, sIFE and uIFE detection indexes, the result shows that: 1 patient in free light chain lambda type, 4 patients in IgA-lambda type and 1 patient in IgG-kappa type. Moreover, 13 (13/143, 9.1%) patients are screened out from 143 patients with normal sFLC ratio.

## DISCUSSION

Multiple researches prove that: the sFLC ratio is known as the important clinical predictive index to indentify the polyclonal plasma diseases and monoclonal plasma diseases [9].

In multiple myeloma, MGUS, solitary plasma-cytoma and lymphoma, and other diseases, the sFLC ratio is believed to have very important function in evaluation disease prognosis [10–12]. The sFLC assay is an important clinical detection measure for assisting to diagnose the light-chain multiple myeloma, non-secreting multiple myeloma and primary amyloidosis [10, 13].

In this clinical research, the result indicates: among 1,340 patients with abnormal sFLC ratio, about 29% patients gets malignant plasma diseases. What is reason causing the patients with non-malignant plasma diseases to get abnormal sFLC ratio? One of the main causes is that, the body is stimulated by all kinds of internal and external factors and results in increased polyclonal immune globulin and its light chain synthesis. Another reason is that, the patient’s combined glomerular disease gives rise to the decreased effective glomerular filtration rate, so as to cause the excretive disorder of immune globulin light chain [14]. In general, the patient with increased polyclonal immune globulin of combined infection or renal function damage may get synchronous increase of serum free light chain kappa and light chain lambda. Therefore, normally, this part of patients’ sFLC ratio is normal or slightly increased. A research has evaluated the normal interval range (0.37-3.1 vs 0.26-1.65) required to be raised in the patients with kidney disease requiring hematodialysis, to detect the monoclonal plasma diseases [15]. Above two reasons are enough to explain that, most of patients with abnormal sFLC ratio gets the reactive polyclonal plasmacytosis, such as autoimmune disease, chronic kidney and infection, etc.

Singh et al. have reported that, the detection method by abnormal sFLC ratio to identify monoclonal plasma diseases has very high false positive rate [8]. In this research, if judging according to the normal interval range (0.26-1.65) of traditional sFLC ratio, about 36.4% patients with abnormal sFLC ratio shall be diagnosed to not belong to the monoclonal plasma diseases. If judging by normal interval range (0.37-3.1) of sFLC ratio after amending the renal damage, about 30.1% patients with abnormal sFLC ratio shall be diagnosed to not belong to the monoclonal plasma diseases. The relevant researches believe that, the abnormal sFLC ratio cannot be deemed as the only index for diagnosing the malignant plasma diseases [16, 17].

Singh, Gurmukh et al. reported that the performance of abnormal sFLC *k*/λ ratio in patients without monoclonal gammopathies had high false-positive rate in the American journal of clinical pathology [8]. Thus, an abnormal sFLC κ/λ ratio should not be considered as the sole indicator for diagnosis of neoplastic proliferation of the lymphoplasmacytic system [18, 19]. In their observations, using the conventional sFLC *k*/λ ratio, 36.4% of the ratios were abnormal without monoclonal gammopathies. When the renal sFLC *k*/λ ratio was used, the rate of abnormal sFLC *k*/λ ratios was 30.1%.

The research shows, in 1,340 patients with abnormal sFLC ratio, the malignant plasma disease occupies 29.0% and MGUS occupies 4.6%. If judging the normal interval range (0.26-1.65) of traditional sFLC ratio the sensitivity and specificity of sFLC assay respectively are 85.27% and 78.02%. Artur Jurczyszyn and other scientists’s research show: the abnormal sFLC ratio may not always be able to identify the monoclonal plasma diseases [19]. However, the International myeloma working group has not defined the new threshold value of abnormal sFLC ratio to improve the sensitivity and specificity for diagnosing monoclonal plasma diseases.

So far, there is no similar article to specifically evaluate, discuss and improve the sensitivity and specificity of abnormal sFLC ratio to diagnosing the monoclonal plasma diseases. In this clinical study, we try to find a better diagnosis point to solve the above problems.

**Figure 3:**
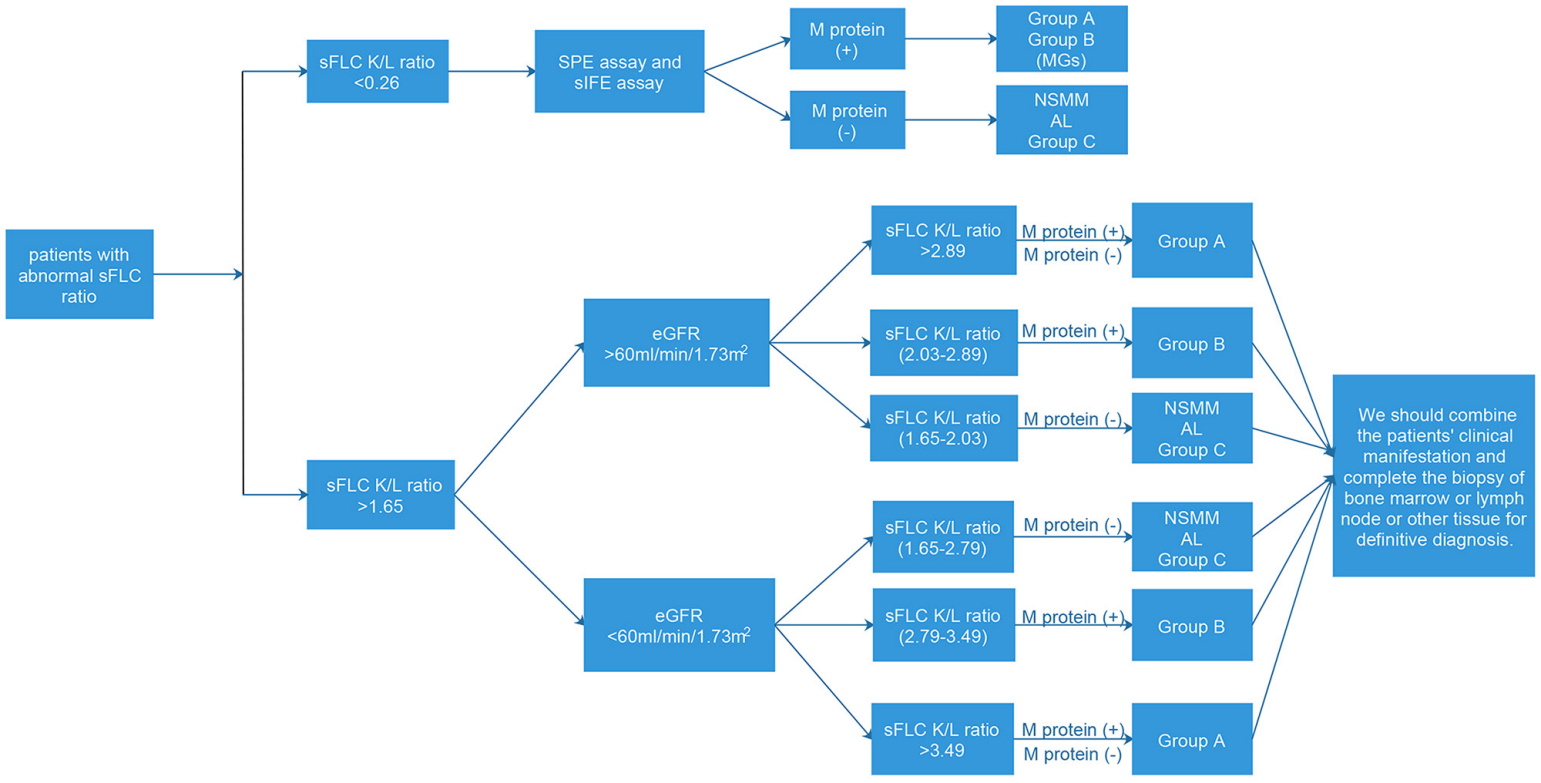
Flow chart of diagnosis of diseases with abnormal sFLC ratio Group A, malignant plasma diseases; Group B, MGUS; Group C, reactive plasma diseases; MGs, monoclonal gammopathies; AL, primary amyloidosis; NSMM, non-secretory multiple myeloma; M protein (+), SPE assay or sIFE assay shows M protein is positive; M protein (-), both SPE assay and sIFE assay show M protein are negative.

Firstly, via the large-sample clinical retrospective study data, determine the disease composition spectrum of abnormal sFLC ratio, and enable the clinical physician to know that, the disease spectrum of abnormal sFLC ratio includes the malignant plasma diseases, MGUS and reactive plasma diseases. The reactive plasma diseases comprise the chronic kidney disease, autoimmune disease, infection, non-lymphoid neoplasia, peripheral neuropathy, myocardiopathy and other benign diseases. In order to improve the correct diagnosis rate of monoclonal plasma diseases and provide the feasible clinical guidance for the clinical physician, we find the new diagnosis point of sFLC ratio for malignant plasma diseases by statistics method (ROC curve). For the purpose of eliminating the definition of renal damage to the sFLC ratio, we divide 1,340 patients into two groups (eGFR>60ml/min/1.73m^2^; eGFR<60ml/min/1.73m^2^). According to the displayed result of Figure 2, when the patient’s sFLC ratio is abnormal, we firstly consider the patient’s renal function, and suggest evaluating with renal function index eGFR. In this study, we once tried to make summary, statistic and analysis on the evaluation index of renal function in accordance with eGFR <90 ml/min/1.73m^2^ or eGFR >90 ml/min/1.73m^2^, and found the sensitivity and specificity of critical value of redefined light chain ratio to diagnose the malignant plasma diseases both are lower than the result summarized and analyzed according to eGFR <60 ml/min/1.73m^2^ or eGFR >60 ml/min/1.73m^2^. Therefore, it has objective basis of data analysis for us to define the critical threshold value eGFR of renal function damage at 60 ml/min/1.73m^2^. When the patient’s eGFR >60ml/min/1.73m^2^ and sFLC κ/λ ratio >2.89, or the patient’s eGFR <60ml/min/1.73m^2^ and sFLC κ/λ ratio >3.49, it is more likely to consider the patient with combined monoclonal plasma diseases, while certainly, it requires further combining with patient’s clinical manifestation, SPE, sIFE, bone marrow cytology, bone marrow or local tissue biopsy to confirm the diagnosis. When the patient’s eGFR >60ml/min/1.73m^2^ and 2.03< sFLC κ/λ ratio <2.89, or the patient’s eGFR <60ml/min/1.73m^2^ and 2.79< sFLC K/L ratio <3.49, it is more likely to consider the patient with combined MGUS, and it requires further combining with patient’s clinical manifestation, SPE, sIFE, bone marrow cytology, bone marrow or local tissue biopsy to confirm the diagnosis. In order to more visually describe and analyzing the grading diagnosis process of abnormal sFLC ratio, see Figure 3 for the details.

The clinical manifestations of relevant diseases, including mild injury and progressive, malignant tumorous diseases, for sFLC ratio abnormality are various. Especially, MGUS and primary systematic amyloidosis diseases are easily misdiagnosed [20] as the result of relatively latent clinical manifestations and low morbidity. In our study, three main comorbidities of MGUS include renal disease, infection and autoimmune disease. According to relevant studies, it has been confirmed that MGUS patients with abnormal sFLC ratio has the higher risk of disease progression than these with normal sFLC ratio. If three risk factors of MGUS patients, including abnormal sFLC ratio, non-IgG MGUS and serum M protein >15g/L, are combined, for about 58% of patients, such disease will be developed into the malignant plasma diseases after 20 years. If 2 risk factors, 1 risk factor and 0 risk factor are respectively combined, the proportions of number of patients with disease progression will be 37%, 21% and 5% after 20 years [10]. In the future clinical studies, we will pay more attention to the relationship between MGUS and its comorbidities.

Among the 15 amyloidosis patients with abnormal combined sFLC ratio, they are mainly light-chain lambda type, and these patients mainly show renal and cardiac damages. The conclusion is consistent with that published in American Journal of Hematology in March, 2015. In the paper, it also indicates that the level of sFLC ratio is an important prognostic factor for evaluation on primary systematic amyloidosis [21]. At present, the studies on the importance of molecular biology abnormality of amyloidosis patients are relatively few compared with these of other plasma diseases. In the AL patients with normal sFLC ratio, the deficiency of No. 13 chromosome could predict the overall response rate (OR) of treatment on patients, but not evaluate the overall survival rate (OS) of patients [22]. In future studies, we will further investigate the association between plasmocyte FISH detection marker and prognosis of AL patient.

POEMS patients with abnormal sFLC ratio are mainly light-chain lambda type. The number of POEMS patients with normal sFLC ratio (13 cases) is significantly higher than these with abnormal sFLC ratio (6 cases). Therefore, POEMS syndrome shall be definitely diagnosed with sFLC assay, SPE, sIFE and patients’ clinical manifestations. For extremely low concentration of monoclonal immunoglobulin or light chain of POEMS patients, it will not perfectly used for monitoring of disease activity. Therefore, the sFLC ratio has a certain limitation in diagnosis and treatment guidance and prognosis evaluation of POEMS patients [23].

In conclusion, the abnormal sFLC ratio most frequently shows monoclonal plasma diseases, but it also contains a broad spectrum of diseases such as reactive plasma diseases. Combining the new cut-off value of sFLC *k*/λ ratio and renal function eGFR can improve the sensitivity and specificity of sFLC assay for diagnosing monoclonal plasma diseases.

## MATERIALS AND METHODS

### Patients

This study incorporates a total of 4,786 cases with complete set of myeloma detection in preliminary diagnosis at Wuhan Tongji Hospital from September, 2012 to June, 2016. The complete set of myeloma detection contains the sFLC assay, serum protein electrophoresis (SPE), serum immunofixation electrophoresis (sIFE) and urine immunofixation electrophoresis (uIFE), etc. This part of patients have been admitted in Wuhan Tongji Hospital mainly due to one or many accompanied clinical manifestations of anemia, increased seroglobulin, skeleton pain, positive urine protein, abnormal renal function, peripheral neuropathy of four limbs and myocardial hypertrophy, and all have accepted the sFLC assay. Among them, 1,340 patients with combined abnormal sFLC ratio are screened out. Further collect the datum of clinical cases, including the gender, age, clinical manifestation, data of complete set of myeloma and patient’s definite diagnosis. Moreover, among 4,786 cases, 143 patients with combined normal sFLC ratio are screened out, with positive M protein qualitative (sIFE) and/or M protein quantitative (SPE) detection.

With sFLC reagent, buffering agent and quality control liquid supplied by FreeliteTM of UK Binging Site, use the Behring BNII full-automatic turbidity meter, make parameter setting according to the reagent specification, carry out the sFLC kappa and lambda concentration detection by immunoturbidimetry after acquiring the ideal calibration, and then calculate the sFLC ratio. SPE, sIFE and uIFE detections are made with full-automatic electrophoresis apparatus. The assay reported the sFLC k/λ ratio (diagnostic range 0.26-1.65), and the results of sFLC k/λ ratio that were out of range were defined as abnormal sFLC k/λ ratio.

Diagnosis of 1,340 screened patients with abnormal sFLC ratio includes the multiple myeloma (MM), plasma cell leukemia (PCL), Waldenstrom macroglobulinemia/lymphoplasmacytic lymphoma (WM/LPL), solitary plasmacytoma (SP), primary systematic amyloidosis (AL), POEMS syndrome (POEMS), B cell non-Hodgkin’s lymphoma (B-NHL), monoclonal gamnopathy of undetermined significance (MGUS), autoimmune disease (AID), chronic kidney disease (KD), infection/chronic inflammatory condition (I/CIC), non-lymphoid neoplasia (NLN), peripheral neuropathy (PN), heart disease particularly myocardiopathy (HD) and other benign diseases (OBD), etc. Other benign diseases mainly include the cerebrovascular diseases and diseases related to endocrine system (such as cerebral infarction, hyperthyroidism and subacute thyroiditis, etc.). 143 patients with combined normal sFLC ratio are also screened out at the same time, accompanied by positive M protein qualitative (sIFE) and/or M protein quantitative (SPE) detection. These 143 patient diagnoses comprise MM, WM/LPL, SP, POEMS, AL, B-NHL and MGUS. The diagnosis standard of above monoclonal plasma diseases is in accordance with NCCN diagnosis guideline in 2016 [4–7].

Divide 1,340 patients with abnormal sFLC ratio into 3 groups: Group A: malignant plasma diseases; Group B: low-malignant-potential (monoclonal gammopathy of undetermined significance, MGUS); Group C: reactive plasma diseases. Diseases in Group A include MM, PCL, WM/LPL, SP, POEMS, AL and B-NHL; disease in Group B mainly is MGUS; diseases in Group C mainly include AID, KD, I/CIC, NLN, PN, HD and OBD. In order to eliminate the influence of renal function injury on the new threshold value of sFLC ratio, we further divide 1,340 patients into two large groups according to the difference of effective glomerular filtration rate (eGFR): 1.eGFR>60ml/min/1.73m^2^; 2. eGFR<60ml/min/1.73m^2^.

### Statistical analysis

The median and interval range are applied to describe and analyze the continuous numerical variable (abnormal distribution datum). X^2^ detection is applied to analyze the qualitative data information. The non-parametric test (Kruskal-Wallis test) is adopted to compare the sFLC ratio difference among the Group A, B and C. Redefine the new threshold value of sFLC ratio by utilizing the ROC curve (receiver operating characteristic curve) to diagnose the malignant plasma cell disease, ROC curve is the curve drawn with true positive rate (sensitivity) as ordinate and false positive rate (1-specificity) as abscissa. Find the optimal diagnosis point by calculating the area under the curve, to find the maximum area under the curve and enable the diagnosis test sensitivity and specificity to reach the optimal state. All incorporated case data information are statistically analyzed by applying SPSS22 software, with result of P<0.05 being believed to have statistical significance.

## Abbreviations

MM: multiple myeloma
PCL: plasma cell leukemia
WM: Waldenstrom macroglobulinemia
LPL: lymphoplasmacytoid lymphoma
SP: solitary plasmacytoma
POEMS: POEMS syndrome
AL: primary amyloidosis
B-NHL: B cell non-Hodgkin’s lymphoma
MGUS: monoclonal gammopathy of undetermined significance
AID: autoimmune disease
KD: kidney disease
I/CIC: Infection/chronic inflammatory condition
NLN: Non-lymphoid neoplasia
PN: peripheral neuropathy
HD: heart disease
OBD: other benign diseases
MGs: monoclonal gammopathies
eGFR: effective glomerular filtration rate
sFLC: serum free light chain
rFLC: the ratio of sFLC

## References

[1] Giarin MM, Giaccone L, Sorasio R, Sfiligoi C, Amoroso B, Cavallo F, Cipriani A, Palumbo A, Boccadoro M (2009). Serum free light chain ratio, total kappa/lambda ratio, and immunofixation results are not prognostic factors after stem cell transplantation for newly diagnosed multiple myeloma. Clin Chem.

[2] Snozek CL, Katzmann JA, Kyle RA, Dispenzieri A, Larson DR, Therneau TM, Melton LJ, Kumar S, Greipp PR, Clark RJ, Rajkumar SV (2008). Prognostic value of the serum free light chain ratio in newly diagnosed myeloma: proposed incorporation into the international staging system. Leukemia.

[3] Guenet L, Decaux O, Lechartier H, Ropert M, Grosbois B (2007). Usefulness of a free light chain immunoassay in serum for the diagnosis and the follow-up of monoclonal gammopathy. Rev Med Interne.

[4] Gertz MA (2015). Waldenstrom macroglobulinemia: 2015 update on diagnosis, risk stratification, and management. Am J Hematol.

[5] Hematology Oncology Committee of China Anti-Cancer Association, Leukemia & Lymphoma Group Society of Hematology at Chinese Medical Association, Union for China Lymphoma Investigators (2016). [The consensus of the diagnosis and treatment of lymphoplasmacytic lymphoma/Walderstrom macroglobulinemia in China (2016 version)]. [Article in Chinese]. Zhonghua Xue Ye Xue Za Zhi.

[6] Mateos MV, Landgren O (2016). MGUS and smoldering multiple myeloma: diagnosis and epidemiology. Cancer Treat Res.

[7] Rajkumar SV (2016). Multiple myeloma: 2016 update on diagnosis, risk-stratification, and management. Am J Hematol.

[8] Singh G (2016). Serum free light chain assay and kappa/lambda ratio performance in patients without monoclonal gammopathies: high false-positive rate. Am J Clin Pathol.

[9] Katzmann JA, Kyle RA, Benson J, Larson DR, Snyder MR, Lust JA, Rajkumar SV, Dispenzieri A (2009). Screening panels for detection of monoclonal gammopathies. Clin Chem.

[10] Rajkumar SV, Kyle RA, Therneau TM, Melton LJ, Bradwell AR, Clark RJ, Larson DR, Plevak MF, Dispenzieri A, Katzmann JA (2005). Serum free light chain ratio is an independent risk factor for progression in monoclonal gammopathy of undetermined significance. Blood.

[11] Kyrtsonis MC, Vassilakopoulos TP, Kafasi N, Sachanas S, Tzenou T, Papadogiannis A, Galanis Z, Kalpadakis C, Dimou M, Kyriakou E, Angelopoulou MK, Dimopoulou MN, Siakantaris MP (2007). Prognostic value of serum free light chain ratio at diagnosis in multiple myeloma. Br J Haematol.

[12] Furtado M, Shah N, Levoguer A, Harding S, Rule S (2013). Abnormal serum free light chain ratio predicts poor overall survival in mantle cell lymphoma. Br J Haematol.

[13] Wang PF, Xu Y, Yan S, Yao Y, Zheng HF, Ma L, Jin S, Xu Y, Gong FR, Zhou JZ, Chang HR, Fu CC (2016). [The roles of serum free light chain ratio in the diagnosis and prognosis of newly diagnosed multiple myeloma]. [Article in Chinese]. Zhonghua Xue Ye Xue Za Zhi.

[14] Katzmann JA, Clark RJ, Abraham RS, Bryant S, Lymp JF, Bradwell AR, Kyle RA (2002). Serum reference intervals and diagnostic ranges for free kappa and free lambda immunoglobulin light chains: relative sensitivity for detection of monoclonal light chains. Clin Chem.

[15] Piehler AP, Gulbrandsen N, Kierulf P, Urdal P (2008). Quantitation of serum free light chains in combination with protein electrophoresis and clinical information for diagnosing multiple myeloma in a general hospital population. Clin Chem.

[16] Diamantidis MD, Ioannidou-Papagiannaki E, Ntaios G (2009). Novel extended reference range for serum kappa/lambda free light chain ratio in diagnosing monoclonal gammopathies in renal insufficient patients. Clin Biochem.

[17] Jagannath S (2007). Value of serum free light chain testing for the diagnosis and monitoring of monoclonal gammopathies in hematology. Clin Lymphoma Myeloma.

[18] Cho SY, Nam YS, Yoon HJ, Lee HJ, Park TS (2013). Clinical significance of abnormal serum free light chain ratio: diagnostic confusion or underlying monoclonality?. Clin Lab.

[19] Jurczyszyn A, Ochrem B (2015). Abnormal serum free light chain ratio does not always indicate monoclonal gammopathy. Pol Arch Med Wewn.

[20] Kaufman GP, Dispenzieri A, Gertz MA, Lacy MQ, Buadi FK, Hayman SR, Leung N, Dingli D, Lust JA, Lin Y, Kapoor P, Go RS, Zeldenrust SR (2015). Kinetics of organ response and survival following normalization of the serum free light chain ratio in AL amyloidosis. Am J Hematol.

[21] Hogan JJ, Weiss BM (2016). Bridging the divide: an onco-nephrologic approach to the monoclonal gammopathies of renal significance. Clin J Am Soc Nephrol.

[22] Zhao L, Tian Z, Fang Q (2016). [The prognostic value of baseline serum free light chain in cardiac amyloidosis]. [Article in Chinese]. Zhonghua Nei Ke Za Zhi.

[23] Wang C, Su W, Cai QQ, Cai H, Ji W, Di Q, Duan MH, Cao XX, Zhou DB, Li J (2016). Prognostic value of serum heavy/light chain ratios in patients with POEMS syndrome. Eur J Haematol.

